# Ectomycorrhizal Inoculation Enhances the Salt Tolerance of *Quercus mongolica* Seedlings

**DOI:** 10.3390/plants10091790

**Published:** 2021-08-27

**Authors:** Xiao-Ning Bai, Han Hao, Zeng-Hui Hu, Ping-Sheng Leng

**Affiliations:** 1 Beijing Advanced Innovation Center for Tree Breeding by Molecular Design, College of Landscape Architecture, Beijing University of Agriculture, Beijing 102206, China; XiaoningBai96@163.com (X.-N.B.); HaoHan9664@163.com (H.H.); 2 China Meteorological Press, Beijing 100081, China

**Keywords:** *Quercus mongolica*, ectomycorrhiza, NaCl, stress

## Abstract

Salt stress harms the growth and development of plants, and the degree of soil salinization in North China is becoming increasingly severe. Ectomycorrhiza (ECM) is a symbiotic system formed by fungi and plants that can improve the growth and salt tolerance of plants. No studies to date have examined the salt tolerance of *Quercus mongolica*, a typical ectomycorrhizal tree species of temperate forests in the northern hemisphere. Here, we inoculated *Q. mongolica* with two ectomycorrhizal fungi (*Gomphidius viscidus*; *Suillus luteus*) under NaCl stress to characterize the effects of ECM. The results showed that the symbiotic relationship of *Q. mongolica* with *G. viscidus* was more stable than that with *S. luteus*. The cross-sectional area of roots increased after inoculation with the two types of ectomycorrhizal fungi. Compared with the control group, plant height, soluble sugar content, and soluble protein content of leaves were 1.62, 2.41, and 2.04 times higher in the *G. viscidus* group, respectively. Chlorophyll (Chl) content, stomatal conductance (Gs), and intracellular CO_2_ concentration (Ci) were significantly higher in *Q. mongolica* inoculated with ectomycorrhizal fungi than in the control, but differences in the net photosynthetic rate (Pn), transpiration rate (Tr), and photosystem II maximum photochemical efficiency (Fv/Fm) were lower. The relative conductivity of *Q. mongolica* inoculated with the two ectomycorrhizal fungi was consistently lower than that of non-mycorrhizal seedlings, with the effect of *G. viscidus* more pronounced than that of *S. luteus*. The malondialdehyde (MDA) content showed a similar pattern. Peroxidase (POD) and catylase (CAT) levels in mycorrhizal seedlings were generally higher than those of non-mycorrhizal seedlings under normal conditions, and were significantly higher than those of non-mycorrhizal seedlings on the 36th and 48th day after salt treatment, respectively. Overall, the results indicated that the salt tolerance of *Q. mongolica* seedlings was improved by ectomycorrhizal inoculation.

## 1. Introduction

Salinity causes a series of cellular, metabolic, and physiological changes. Excessive salt in the soil is harmful to the growth and development of plants, as salt stress has both osmotic effects on cells and can lead to ion toxicity. The secondary effects of salt stress are also complex and include: oxidative stress; damage to cell membrane lipids, proteins, nucleic acids, and other cellular components; metabolic disorders [1]. Salt stress can also result in the competitive absorption of Na^+^ and K^+^ by plant roots, thereby affecting normal cellular function [2]. Plants reduce the absorption of Na^+^ and, thus, the upward transport of Na^+^ by regulating the gene expression and protein function of membrane transporters. Plants also limit transpiration by closing stomata and regulating root water absorption, which is mainly driven by the strict regulation of aquaporins [3,4]. To prevent oxidative stress in cells, plants also accumulate compatible osmolytes or osmoprotectants in response to salt stress [5]. Chaves et.al. [6] found that salinity led to a reduction in CO_2_ availability. One of the first responses of plants to salt stress is stomatal closure, which impairs CO_2_ uptake [6]. In addition, a de-activation of the carboxylating enzyme Rubisco by low intercellular CO_2_ has been observed [7]. The supply of CO_2_ to Rubisco is therefore impaired. At the cellular level, salt stress can cause osmotic stress and ion deficiency, damage to cell membranes, decreased chlorophyll content, decreased enzyme activity, and, ultimately, disruption of photosynthesis [6,8]. However, with several previous studies reporting that these responses may not be sufficient for combating prolonged salt stress, further physiological, ecological, and evolutionary adaptations are required.

Many approaches have been used to enhance the salt tolerance of plants. Ectomycorrhizal inoculation has been shown to be a particularly effective approach [9], as several previous studies have reported that ectomycorrhizal associations can protect plants against salt stress [10,11,12,13,14,15,16]. Recently, Zwiazek et al. [17] found that urban ectomycorrhizal fungi could improve the tolerance of *Pinus contorta* seedlings to salt stress. Several strategies have been proposed to enhance the tolerance of plants to salt stress, including restricting the entry of Na^+^ into plant tissues helping to maintain mineral nutrition and water balance [9]. In addition, Otgonsuren et al. [18] found that ectomycorrhiza (ECM) enhanced the chlorophyll content of *Populus nigra* and maintained normal levels of root respiration under saline conditions. Sa et al. [15] demonstrated that ECM contributes to enhanced salt tolerance in *Populus canescens* hybrid lines by helping to maintain NO^3-^ levels in plants during salt stress. Moreover, the stress response pathway (metabolites, hormones) activated by ectomycorrhizal colonization can help alleviate salt stress [14]. However, the exact mechanism by which ectomycorrhizal fungi contribute to alleviating the effects of salinity on their host plants remains unclear [9].

With more than 300 species, the genus *Quercus* is one of the most diverse and widely distributed groups of forest tree species in the world. In China, *Quercus* accounts for 13% of the total area of natural forest, making it the most widely distributed tree species in the country. Thus, *Quercus* plants play an important role in the stability of forest ecosystems. Ectomycorrhizal fungi are important symbionts of the roots of Fagaceae species in boreal and temperate forests, including *Quercus* [19,20]. Previous studies have shown that ectomycorrhizal fungi can improve the growth of *Quercus* plants. Inoculation with *Tuber melanosporum* enhances the net assimilation rate, stomatal conductance, and root conductance per unit leaf surface of *Q. ilex* seedlings [21]. *Q. rubra* seedlings colonized by ectomycorrhizal fungus show enhanced productivity in several respects compared with uncolonized seedlings, including the number of leaves produced, leaf area, and basal stem diameter; colonized seedlings also supported lower caterpillar growth and experienced lower consumption rates [22]. Inoculation with *Gomphidius viscidus* and *Russula foetens* significantly promotes the growth of *Q. liaotungensis* seedlings [23]. Mycorrhization has also been shown to improve seedling growth, total phosphorus absorption, and water uptake of the seedlings during summer drought [24]. However, the effect of ectomycorrhizal fungus on the tolerance of *Quercus* plants to other stress conditions, especially salt stress, remains unknown.

*Q. mongolica* is a tree species, distributed in forests of North China, which has high ecological and ornamental value. *Q. mongolica* is often used in garden landscaping, as its leaves are unique in shape and turn red in autumn. The ability of *Q. mongolica* to tolerate salt stress and its underlying mechanism, especially the role of ECM, have not been studied to date. Here, we tested the hypothesis that inoculation of *Q. mongolica* seedlings by ECM affects their morphology, photosynthesis, and physiology. We inoculated *Q. mongolica* seedlings with either *G. viscidus* or *S. luteus*, grew them under salt-free conditions or in the presence of 120 mM NaCl, and compared the morphology, photosynthesis characteristics, and physiology of these inoculated plants with uninoculated control plants.

## 2. Results

### 2.1. External Morphology and Anatomical Structure of ECM

**Figure 1:**
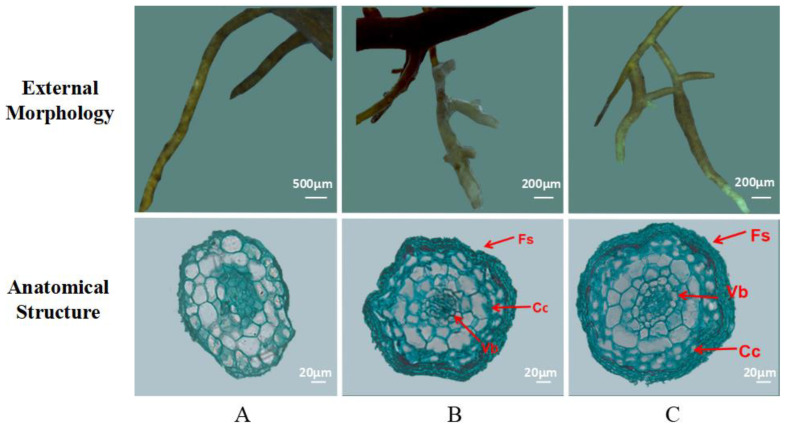
The external morphology and anatomical structure of the mycorrhiza of *Q. mongolica*. Note: (**A**) control group (non-mycorrhizal roots, no salt stress); (**B**) *G. viscidus* group; (**C**) *S. luteus* group. Fs: fungus sheath; Cc: cortical cells; Vb: vascular bundle.

Figure 1 shows the morphology and anatomical structure of the roots of *Q. mongolica* seedlings after inoculation with two ectomycorrhizal fungi. The typical structural characteristics of the ECM were observed. ECM was significantly shorter and thicker than the normal root system; the mycelial mantle, which is white and tawny, is formed by mycelium around the root system. The ECM included the constricting ring and cortical cells. The cross-sectional area of roots in different fungal inoculation groups varied; the cross-sectional area was highest for the *S. luteus* group, followed by the *G. viscidus* group and the control group. As the inoculation time extended, the root cortical cells appeared to occur deeper in the Hartig net. Cortical cells changed in diverse ways under the different fungal inoculations. After inoculation, the cortical cells in *G. viscidus* seedlings began to shrink before those in *S. luteus* seedlings, and the degree of shrinkage was more severe in *G. viscidus* seedlings.

As the inoculation time extended, the root radius, middle column radius, and cortex thickness of the ECM in *Q. mongolica* increased (Table 1). There was no significant correlation between the thickness of the constricting ring with the time of inoculation and with fungal species. The radius of the middle column and the roots in the control group and *S. luteus* seedlings increased continuously, whereas those in *G. viscidus* seedlings decreased significantly. On the 30th day of inoculation, the ratio of the column radius to the root radius in *G. viscidus* seedlings was 0.337, and that of *S. luteus* seedlings was 0.378, which was the highest for the middle column in ECM.

### 2.2. Effect of NaCl Treatment on Growth

**Figure 2:**
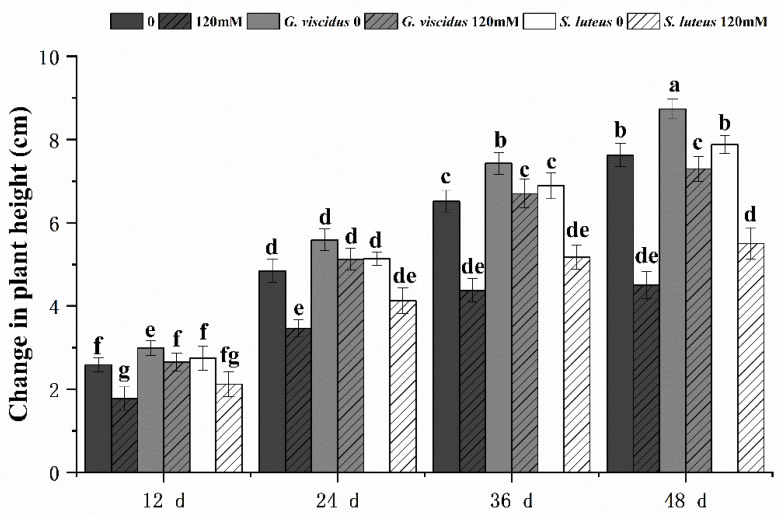
Changes in plant height in ectomycorrhizal seedlings of *Q. mongolica* under NaCl stress. Bars represent means of five replicates (±SD). For each parameter, bars with different letters indicate significant differences among treatments, *p* ≤ 0.05, following one-way ANOVA.

Salt had a negative effect on seedling growth; the degree of growth inhibition (i.e., plant height) was lower in inoculated *Q. mongolica* than in the control. Under the NaCl treatment, the growth of seedlings inoculated with ectomycorrhizal fungi was inhibited to different degrees; inoculation with *G. viscidus* alleviated this negative effect, whereas inoculation with *S. luteus* had virtually no effect (Figure 2). The growth of *G. viscidus* and *S. luteus* seedlings was higher than that of control group seedlings under NaCl stress; the growth of *G. viscidus* seedlings on day 48 under 120 mM stress was 1.52 times higher than that of control group seedlings, whereas that of *S. luteus* seedlings was only 1.15 times higher. The difference in the growth of *S. luteus* seedlings relative to the control group seedlings decreased gradually throughout the experimental period.

### 2.3. Effect of NaCl Treatment on Photosynthesis

**Figure 3:**
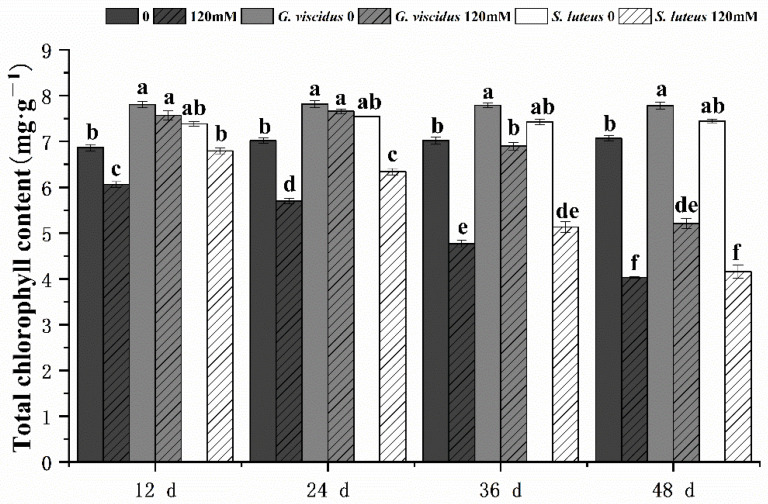
Changes in the total chlorophyll content in ectomycorrhizal seedlings of *Q. mongolica* under NaCl stress. Bars represent means of five replicates (±SD). For each parameter, bars with different letters indicate significant differences among treatments, *p* ≤ 0.05, following one-way ANOVA.

**Figure 4:**
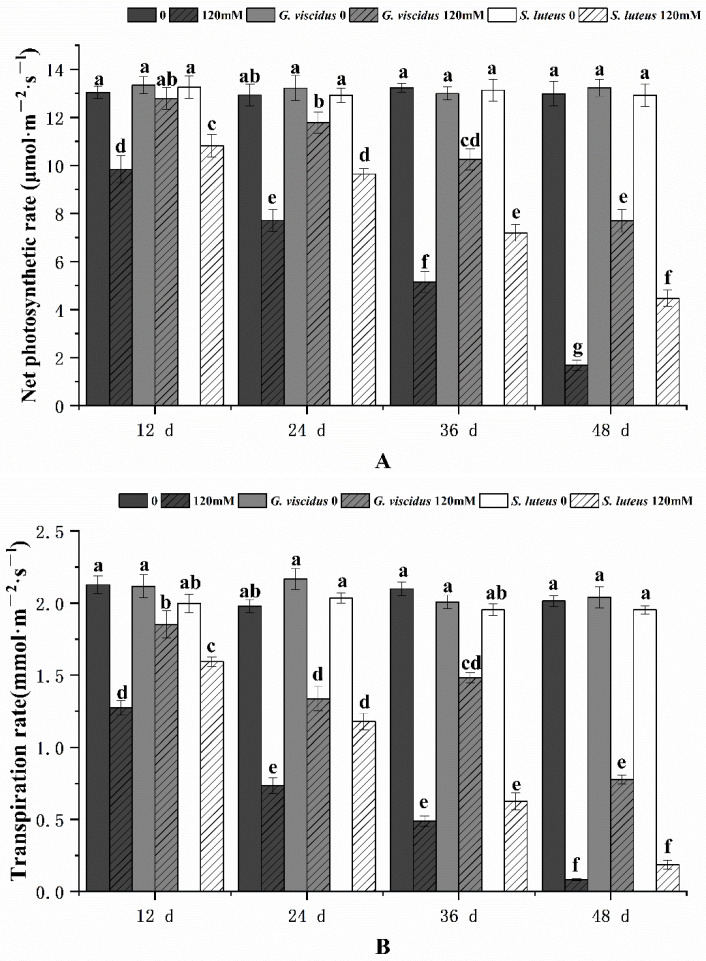
Changes in the net photosynthetic rate (**A**) and transpiration rate (**B**) in ectomycorrhizal seedlings of *Q. mongolica* under NaCl stress. Bars represent means of five replicates (±SD). For each parameter, bars with different letters indicate significant differences among treatments, *p* ≤ 0.05, following one-way ANOVA.

**Figure 5:**
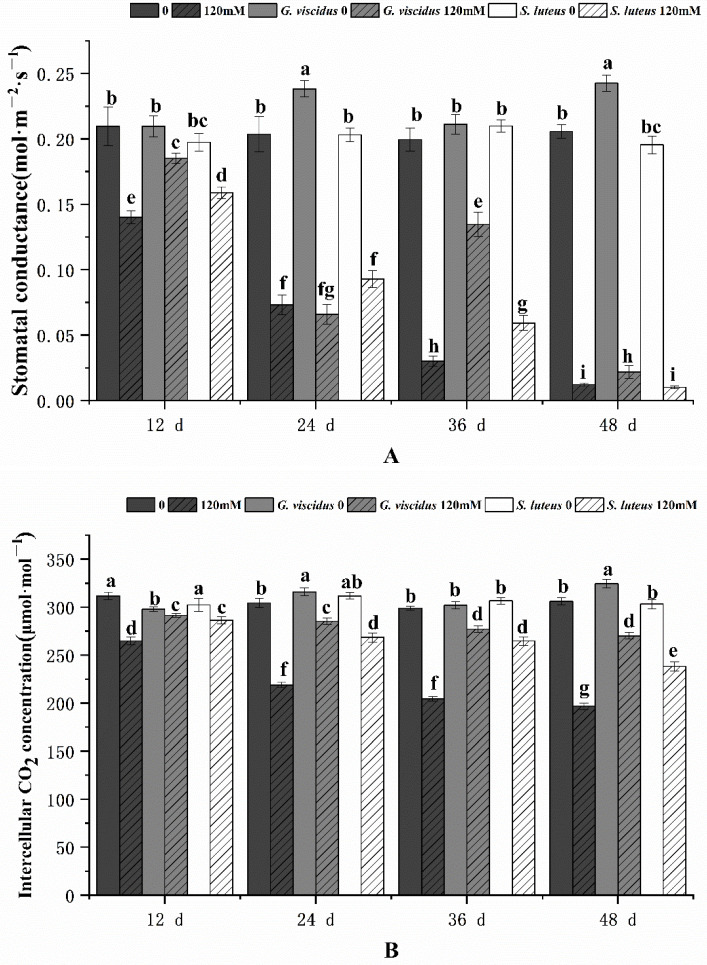
Changes in the stomatal conductance (**A**) and intercellular CO_2_ concentration (**B**) in ectomycorrhizal seedlings of *Q. mongolica* under NaCl stress. Bars represent means of five replicates (±SD). For each parameter, bars with different letters indicate significant differences among treatments, *p* ≤ 0.05, following one-way ANOVA.

The photosynthetic characteristics of mycorrhizal and non-mycorrhizal *Q. mongolica* seedlings were also measured after NaCl treatment. Under salt stress, the Chl content, Pn, Tr, Gs, and Ci in seedlings inoculated with ectomycorrhizal fungi were significantly increased, and these parameters were higher for *G. viscidus* inoculated seedlings than for *S. luteus* inoculated seedlings. Under long-term salt stress, all the photosynthetic indexes of mycorrhizal seedlings decreased, with the exception of Gs and Tr of *G. viscidus* seedlings, which increased on day 36. All indicators for the *G. viscidus* and *S. luteus* seedlings differed the most from control group seedlings at day 36 or 48 under salt stress. The Chl (Figure 3), Pn (Figure 4A), Tr (Figure 4B), Gs (Figure 5A), and Ci (Figure 5B) contents on day 48 of 120 mM stress were 1.29, 4.59, 9.38, 1.80, and 1.37 times higher in *G. viscidus* seedlings than in control group seedlings but 1.03, 2.67, 2.24, 0.83, and 1.21 times higher in *S. luteus* seedlings than in control group seedlings, respectively.

### 2.4. Effect of NaCl Treatment on Chlorophyll Fluorescence Parameters

**Figure 6:**
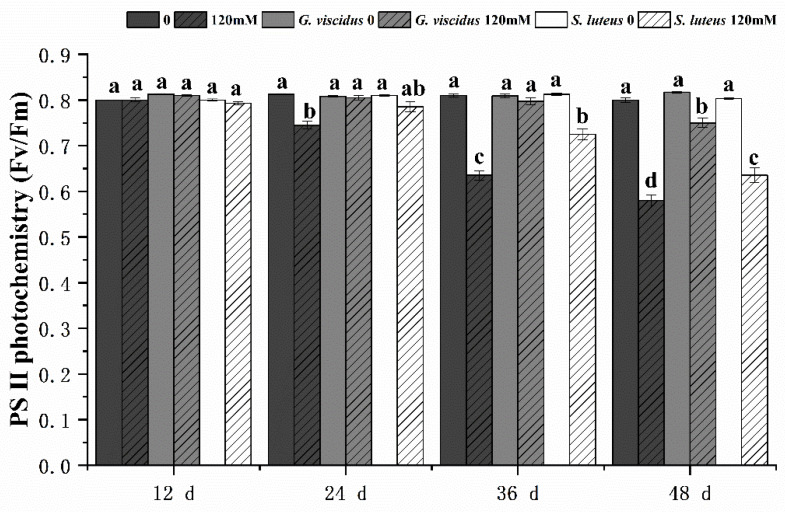
Changes in PSⅡ photochemistry (Fv/Fm) in ectomycorrhizal seedlings of *Q. mongolica* under NaCl stress. Bars represent means of five replicates (±SD). For each parameter, bars with different letters indicate significant differences among treatments, *p* ≤ 0.05, following one-way ANOVA.

Under pre-salt stress, Fv/Fm (Figure 6) showed little change, but at day 36 and 48, Fv/Fm had changed significantly in *G. viscidus* and *S. luteus* seedlings compared with control group seedlings. In addition, Fv/Fm was significantly higher in *G. viscidus* seedlings than in *S. luteus* seedlings. Fv/Fm on day 48 of 120 mM stress was 1.29 times and 1.09 times higher in *G. viscidus* and *S. luteus* seedlings than in control group seedlings, respectively.

**Figure 7:**
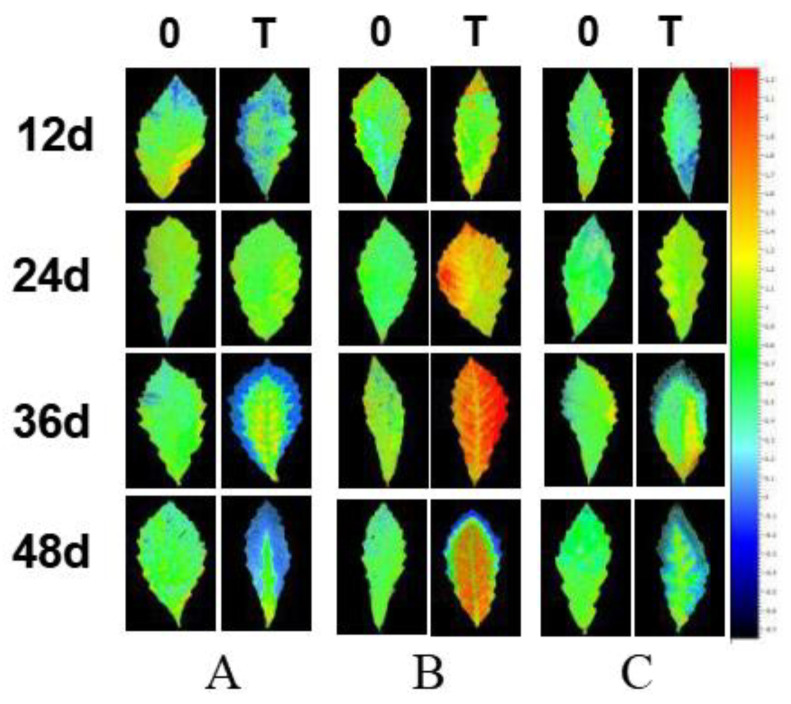
Changes in the non-photochemical quenching (NPQ) in ectomycorrhizal seedlings of *Q. mongolica* under NaCl stress (0: 0 mM; T: 120 mM; (**A**) control group; (**B**) *G. viscidus* group; (**C**) *S. luteus* group).

Under salt stress, changes in the NPQ in different inoculation groups differed. The yellow on the leaf margins of the seedlings became more pronounced, and the NPQ of the necrotic leaf edge was reduced to 0, which is blue in Figure 7. Overall, the NPQ of the leaf margin of each inoculation group decreased, and the blue area gradually increased. *G. viscidus* seedlings showed the smallest change, and the blue area was the largest in control group seedlings, followed by *S. luteus* and *G. viscidus* seedlings. Healthy mesophyll in *S. luteus* seedlings were generally yellow/green, and there was no significant difference in the color compared with control group seedlings. In *G. viscidus* seedlings, the internal leaves turned orange/yellow to red; the NPQ was greater than 1.5 and increased continuously as the stress increased to approximately 2.1, which was significantly higher compared with control group seedlings. NPQ could protect the photosystem from the damage of superfluous luminous energy via heat dissipation. Chloroplasts in leaves became injured under salt stress, the ability of chloroplasts to regulate and protect PSII was weakened, and NPQ decreased. *G. viscidus* might protect chlorophyll and increase the NPQ in the leaves of *Q. mongolica* to a certain extent. Under salt stress, *Q. mongolica* inoculated with *G. viscidus* might improve the NPQ of healthy leaves to make up the NPQ lost in damaged leaves and thus protect the photosystem.

### 2.5. Effect of NaCl Treatment on Cell Membrane Permeability

**Figure 8:**
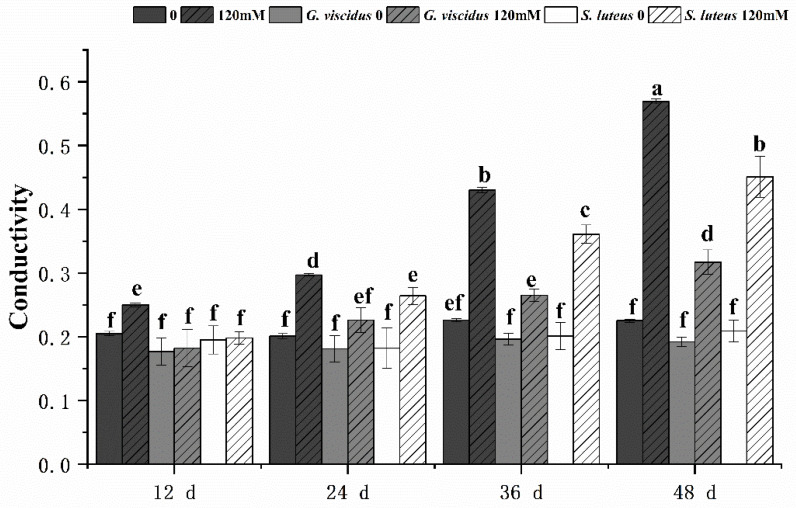
Changes in the relative conductivity in ectomycorrhizal seedlings of *Q. mongolica* under NaCl stress. Bars represent means of five replicates (±SD). For each parameter, bars with different letters indicate significant differences among treatments, *p* ≤ 0.05, following one-way ANOVA.

**Figure 9:**
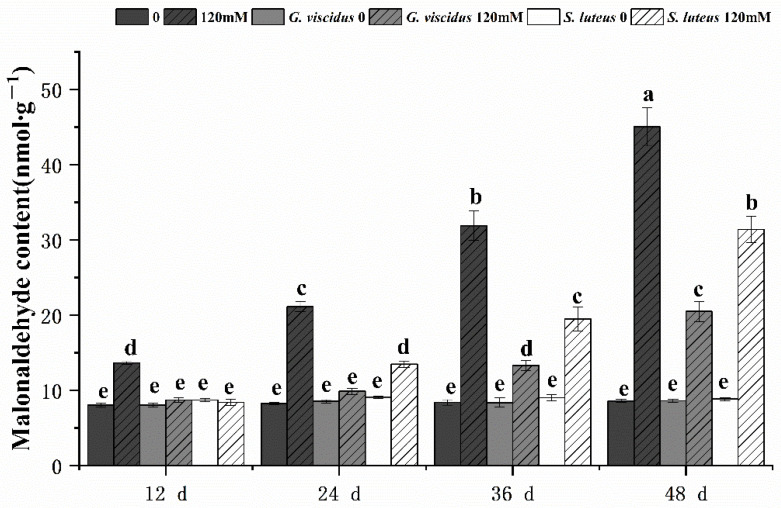
Changes in the malonaldehyde content in ectomycorrhizal seedlings of *Q. mongolica* under NaCl stress. Bars represent means of five replicates (±SD). For each parameter, bars with different letters indicate significant differences among treatments, *p* ≤ 0.05, following one-way ANOVA.

Under NaCl stress, the cell membrane permeability in the leaves of *G. viscidus*, *S. luteus*, and control group seedlings was compared by measuring the relative conductivity (Figure 8) and MDA content (Figure 9). Both indicators increased continuously as the stress duration increased. Compared with the control group, both indexes of the mycorrhizal seedlings were lower at the same time points, and their rates of increase were lower. The patterns of change in the indexes of the seedlings inoculated with different ectomycorrhizal fungi varied; specifically, the relative conductivity and MDA content were significantly lower for *G. viscidus* seedlings than for control group seedlings at all time points, and the relative conductivity and MDA content were 0.56 and 0.46 times higher than those in control group seedlings at day 48 of 120 mM stress, respectively. The two indexes were significantly increased at day 48 of stress in *S. luteus* seedlings compared with control group seedlings, and the relative conductivity was 1.25 times higher in *S. luteus* seedlings than in control group seedlings at day 36.

### 2.6. Effect of NaCl Treatment on Osmotic Adjustment Substances

**Figure 10:**
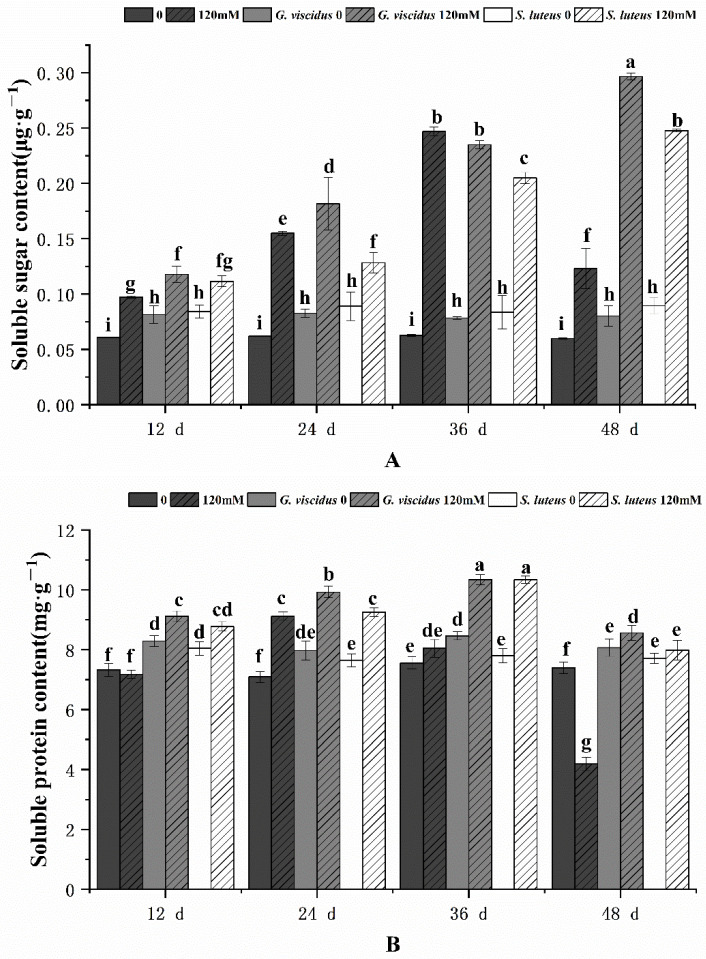
Changes in soluble sugar (**A**), soluble protein (**B**) in ectomycorrhizal seedlings of *Q. mongolica* under NaCl stress. Bars represent means of five replicates (±SD). For each parameter, bars keyed with different letters indicate significant differences among treatments, *p* ≤ 0.05, following one-way ANOVA.

Figure 10 shows the effect of NaCl treatment on sugar content and soluble protein. As the stress duration increased, the soluble sugar content and soluble protein content in *G. viscidus*, *S. luteus*, and control group seedlings initially increased but decreased when the stress was a continuous increase; the exception was for the soluble sugar content in *G. viscidus* and *S. luteus* seedlings, which continually increased and never decreased. ECM enhanced the increase in soluble sugar and soluble protein in seedlings in the pre-stress period, and the greatest increase was observed in *G. viscidus* seedlings. The soluble sugar content was 2.41 and 2.01 times higher in *G. viscidus* and *S. luteus* seedlings than in control group seedlings at day 48 of 120 mM stress, respectively. The soluble protein content was 2.04 and 1.91 times higher in *G. viscidus* and *S. luteus* seedlings than in control group seedlings at day 48, respectively.

### 2.7. Effect of NaCl Treatment on the Antioxidant Enzyme System

**Figure 11:**
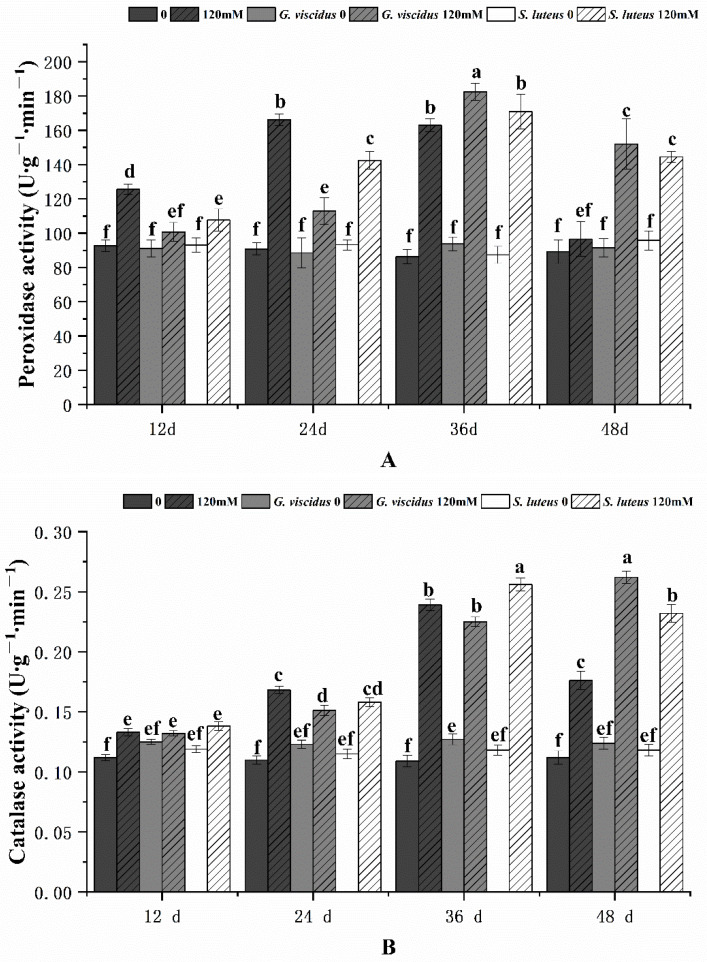
Changes in the POD (**A**), CAT (**B**) activity in ectomycorrhizal seedlings of *Q. mongolica* under NaCl stress. Bars represent means of five replicates (±SD). For each parameter, bars with different letters indicate significant differences among treatments, *p* ≤ 0.05, following one-way ANOVA.

As the stress duration increased, the activities of POD and CAT in *G. viscidus*, *S. luteus*, and control group seedlings first increased and then decreased; the exception was for CAT activity in *G. viscidus* seedlings, which continually increased (Figure 11). The POD activities of *G. viscidus* and *S. luteus* seedlings were highest on day 36, which were 1.12 times and 1.04 times higher compared with control group seedlings, respectively. The CAT activities in *G. viscidus* and *S. luteus* seedlings were highest at day 48 and 36, which were 1.49 times and 1.07 times higher compared with control group seedlings, respectively.

## 3. Discussion

Inoculation with ectomycorrhizal fungi is reported to be an effective approach for enhancing plant salt tolerance [9,11,14,15,17]. *Castanea mollissima* and *Q. mongolica* are Fagaceae plants. Previous studies have shown that most of the ectomycorrhizal symbioses species of *C. mollissima* belong to Basidiomycota [25], which includes *G. viscidus and S. luteus*. *S. luteus* has a wide distribution and has been studied in many Pinaceae plants [26,27,28]; less research has been conducted on *G. viscidus*. However, neither of these two types of ectomycorrhizal fungi have been thoroughly studied in *Quercus*. In *Q. mongolica*, an important tree species in the forests of North China, the role of ECM in salt tolerance has not been studied. In this study, we inoculated *Q. mongolica* seedlings with two ectomycorrhizal fungi, *G. viscidus* and *S. luteus*, and studied their salt tolerance relative to a control inoculated with sterile solid inoculant.

The affinity of the root system of the same host can differ among different strains, which indicates that the host has different degrees of mycorrhizal dependence on different strains [29]. *G. viscidus* can invade the cortical cells of the roots of seedlings more rapidly and form a symbiotic structure. This indicates that *G. viscidus* can more effectively promote the mycorrhization of seedlings. The Hartig net is considered an important indicator of ECM formation [30]. Krause et al. [31] found that indole-3-acetic acid of fungal origin acts as a diffusible signal that increases Hartig net formation in ECM. The two ectomycorrhizal fungi-inoculated seedlings of *Q. mongolica* invaded the two layers of cortical cells with a Hartig net, with no significant difference in the degree of Hartig net formation. This may be the result of the comprehensive effect of host plant age, soil fungus diversity, and effector secreted by the fungus [32].

Salt stress inhibits plant growth [33], and inoculation with ectomycorrhizal fungi can alleviate the adverse effects of various abiotic stresses on plant growth [9]. The ECM can improve the growth of host plants in high-salt environments [34]. Wen et al. [35] found that ectomycorrhizal fungi inoculations improved the salt tolerance of *Pinus thunbergii* to different degrees. The effect of *Laccaria gomezii* was stronger than that of *Cenococcum geophilum*. In this study, we found that ectomycorrhizal fungi significantly promoted the growth of *Q. mongolica*. *G. viscidus* seedlings had the largest increase in plant growth under salt stress; there are several possible explanations for this observation. Some mycorrhizal symbionts mainly regulate soil pH value by secreting hydrogen ions and organic acids to reduce the damage of weak bases to plants [36]. Mycorrhiza may effectively increase the absorption of water [37]. Mycorrhizal extraradical mycelium can alter the surrounding soil to enhance the acquisition of water and nutrients [38]. The hyphal contribution to water absorption in mycorrhizal plants may be affected by the fungal species and water status [37]. Additionally, ECM had greater antioxidant enzyme activity relative to non-mycorrhizal roots, an effect that reduced cellular damage and increased plant growth by preventing ROS formation and ROS detoxification [39].

Several studies have shown that salt stress can lead to complex physiological and biochemical reactions in plants, including osmotic stress, ion toxicity, and oxidation-reduction reactions, which can damage the chloroplasts of leaves [40,41]. Under salt stress, plant chloroplasts tend to disintegrate, and the synthesis of chlorophyll and carotenoids is inhibited. Previous studies have shown that ectomycorrhizal fungi can increase the Chl content of *P. thunbergii* seedlings under salt stress [42]. In our study, the Chl content was higher in mycorrhizal *Q. mongolica* seedlings than in control group seedlings under NaCl treatment, and the decrease in the Chl content became less pronounced as the duration of stress increased, indicating that the chloroplasts of the mycorrhizal seedlings were less damaged. This is similar to the change in Chl content observed in the needles of *P. thunbergii* inoculated with *C. geophilum* under salt stress [42].

As the stress duration increased, Pn and Gs of *Q. mongolica* decreased significantly, and the decrease in Pn became more pronounced, which may be caused by multiple factors. Previous studies have shown that under mild salt stress, the decrease in Pn in plants is related to the Chl content; under severe stress, the decrease in Pn stems from the combined effect of Chl content, Gs, and Ci [43]. The decrease in Pn in seedlings may be related to the change in Chl and Gs. Under salt stress, Gs declined, and stomatal closure was aggravated, which ultimately led to a substantial decrease in Pn under severe salt stress. This pattern was similar to that observed in cotton plants [44]. As the stress duration in *Q. mongolica* increased, Gs decreased, and the degree of stomatal closure increased. Under salt stress, ectomycorrhizal fungi alleviated the stomatal restriction of *Zelkova serrata* and enhanced transpiration, and the decrease in Pn was less pronounced [45]. Both ectomycorrhizal fungi significantly increased the Pn and Gs of seedlings with respect to the control, which was consistent with results obtained for *Z. serrata* [45].

Under salt stress, the NPQ of *Q. mongolica* decreased in the leaf margin and remained unchanged in the central part of the blade. The edge of the leaf was scorched and withered, which reduced heat consumption. *Moms alba* can increase the heat dissipation of energy by increasing NPQ to protect the photosynthetic system from damage [46]. Our study showed that *G. viscidus* and *S. luteus* can protect the photosynthetic system of *Q. mongolica* under stress, and *G. viscidus* had a stronger effect. This result was similar to that observed in *Populus cathayana* [47]. Under salt stress, ectomycorrhizal fungi can increase plant chlorophyll fluorescence efficiency and alleviate the inhibition of plant photosynthesis by salt stress [11]. The inoculation of *Q. mongolica* with *G. viscidus* may facilitate salt adaptation. However, the heat dissipation of *S. luteus* seedlings remained unchanged, which could still protect the photosynthetic system of seedlings to a certain extent under stress.

The relative conductivity and MDA content can reflect the degree of plant cell membrane damage. As the duration of salt stress increased, the relative conductivity and MDA content of *Q. mongolica* increased, indicating greater damage to the cell membrane. In this study, the MDA content was lower in *Q. mongolica* seedlings inoculated with two types of ectomycorrhizal fungi than in non-mycorrhizal seedlings under salt stress. The ECM significantly reduced root hydraulic conductance in *Ulmus americana* but did not affect leaf hydraulic conductance and net photosynthesis under NaCl stress [13]. This indicates that inoculation with ectomycorrhizal fungi can inhibit the production of membrane lipid peroxides and reduce the electrolyte extravasation of seedlings under salt stress. A relative conductivity of more than 50% is a sign of lethal damage to plants. Compared with non-ectomycorrhizal seedlings, the relative conductivity of ectomycorrhizal seedlings does not exceed 50%, which protects plants from damage.

The accumulation of osmotic adjustment substances is positively correlated with the degree of salt tolerance in plants [48]. Under salt stress, *Malus halliana* accumulates soluble sugar to alleviate salt damage and improve salt tolerance [49]. Under long-term stress, the cell membrane was seriously damaged, and the osmotic balance was gradually disrupted. Sharifi et al. [50] found that the proline content in soybean was significantly increased after mycorrhizal fungi inoculation. We found that two types of ectomycorrhizal fungi promoted the accumulation of soluble sugar and soluble protein in *Q. mongolica* pre-stress and enhanced the salt tolerance of seedlings by delaying the onset of osmotic imbalance under long-term stress.

Plants can remove free radicals produced under salt stress to enhance protection from oxidative stress by increasing the activity of POD, CAT, and SOD [48]. As salt stress increases, the antioxidant enzymes of *Q. dentata* first increase and then decrease [51]. This pattern is consistent with our results. Ghorhanli et al. [52] found that mycorrhizal plants have higher SOD and POD activity, and that mycorrhizal plants treated with salt have higher SOD and POD activity than those not treated with salt. This may stem from the fact that ectomycorrhizal fungi can enhance the salt tolerance of *Q. mongolica* by increasing antioxidant activity and delaying enzyme inactivation to adapt to long-term stress.

## 4. Materials and Methods

### 4.1. Plant Material

The seeds of *Q. mongolica* were collected from Beijing Pinggu Glass Valley Forest Farm, soaked with 0.5% KMnO_4_ for 1 h, washed with clean water, and germinated in sand in a greenhouse. The sand substrate was river sand sterilized by high-pressure steam (121 °C, 20 min). After the radicle broke through the seed coat and measured 5 cm, the axial root of the seed was retained and the lateral root was cut to promote germination. The soil matrix ratio was peat–perlite–vermiculite–organic fertilizer = 4:2:1:1.

### 4.2. Preparation of Fungal Inoculant and Fungal Inoculation

Two types of fungi purchased from Chinese strain storage, *G. viscidus* and *S. luteus*, were cultured with potato dextrose agar medium (PDA). Small fruiting bodies were selected and inoculated into a conical bottle containing PDA. After oscillating at a constant temperature for 1 week, the culture fluid was filtered and rinsed with distilled water, which finally gave the solid inoculant.

Fungi inoculation and ectomycorrhizal synthesis were carried out on the seedlings when the seedlings had two leaves. For each pot of seedlings, inoculations were conducted at three points equidistant to the root system, and the three points were connected to form an equilateral triangle. Each inoculation point was inoculated with 20 g of solid inoculant, and the inoculation depth was 5–10 cm. The control group was inoculated in the same way but with sterilized solid inoculant (121 °C, 20 min). First-stage lateral root mycorrhizal segments were then collected from healthy seedlings with uniform growth to characterize the morphology and anatomical structure of the ECM.

### 4.3. Salinity Treatment

Mycorrhizal seedlings with the same growth trend were selected for NaCl treatment. A concentration of 120 mM NaCl was applied to seedlings in the experiment. The seedlings were irrigated with 500 mL of NaCl solution prepared with deionized water every 12 days, and the control group was irrigated with an equal amount of deionized water. There were four NaCl applications over 48 days. When a small number of seedlings died, the experiment was stopped. 

### 4.4. Morphological and Anatomical Structure Measurement

The second-stage lateral roots were observed under a ZEISS Axioskop 40 Stereomicroscope. The representative first-stage lateral root segments were selected for photography and then fixed in formaldehyde–acetic acid–ethanol fixative. Paraffin sections were used to observe the anatomical structure of the ECM. The ECM was observed and recorded with a ZEISS Axiocam 506 color optical microscope after dehydration (soaked in 70%, 80%, 90%, and 100% alcohol for 30 min each), development of transparency (soaked in alcohol–xylene (1:1) and twice in xylene for 30 min each), embedding, sectioning, rehydration (soaked in 100%, 95%, 85%, 75%, 50%, and 30% alcohol for 30 min each), and staining. The anatomical structure of five root segments was studied in each inoculation group at different times after inoculation.

### 4.5. Physiological and Biochemical Indicators Measurement

The MDA and soluble sugar content in leaves was determined by the thiobarbituric acid method [53]. Peroxidase and Catalase activity were assayed by guaiacol colorimetric method and ultraviolet absorption method [54]. Soluble protein content [53] was measured by Coomassie brilliant blue G-250.

On days 12, 24, 36, and 48 after the first NaCl application, the leaves were collected. Two g of leaves were placed in a mortar, and small amounts of CaCO_3_, quartz sand, and 3 mL of 95% ethanol were added. After grinding into a fine powder, 10 mL of 95% ethanol was added, and the sample was further ground until the leaves turned white. After 30 min, the leaves were filtered into a 100 mL volumetric flask. The absorbance value of the extract was determined by a UV-2100 ultraviolet-visible spectrophotometer at the wavelengths of 470 nm, 649 nm, and 665 nm, with 95% ethanol used as a blank control. The total chlorophyll (Chl) content was calculated using the formula of the standard curve.

Live leaves were rinsed 3 times with deionized water; the leaves were then wiped to remove surface moisture, avoiding the leaf vein, and punched into 10 pieces. The sample was then placed in a centrifuge tube; after adding 10 mL of deionized water, the sample was placed in a vacuum pump until the blade sank to the bottom. The sample was then removed and left to stand for 4 h. A DDSJ-308A conductivity meter was used to measure the conductivity value S0 of the deionized water and the initial conductivity value S1. The sample was heated in a boiling water bath for 10 min, rinsed with cold water for 10 min; the final conductivity value S2 was then measured. The relative conductivity was calculated according to the formula in a previous study [53].

### 4.6. Photosynthesis Index Measurements

On days 12, 24, 36, and 48 after the first NaCl application, healthy mature functional leaves with the same size and light exposure were selected for instantaneous measurements of photosynthetic parameters using a LI6400 photosynthesis meter. A light level of 1000 µmol photons m^−2^ s^−1^ was supplied by a red–blue LED. The net photosynthetic rate (Pn), transpiration rate (Tr), stomatal conductance (Gs), and intercellular CO_2_ concentration (Ci) of the leaves were determined. The following set of parameters were used during measurements: stomatal flow rate, 500 mL·min^−1^; leaf temperature, 30 ± 1 °C; relative air humidity, 60%; and concentration of CO_2_ in leaf chamber, 400 μmol·mol^−1^.

### 4.7. Chlorophyll Fluorescence Parameter Measurements

On days 12, 24, 36, and 48 after the first NaCl application, healthy mature functional leaves with the same size and light exposure were selected for measurements of chlorophyll fluorescence parameters. After 15 min of dark adaptation, photosystem II maximum photochemical efficiency (Fv/Fm) and the coefficient of photochemical quenching (NPQ) were measured using a FluorCam 800 MF.

### 4.8. Statistical Data Analysis

All data were analyzed by one-way ANOVA using SPSS 13.0. *p* < 0.05 was the threshold for statistical significance. There were five biological replicates for each treatment.

## 5. Conclusions

Inoculation of *G. viscidus* and *S. luteus* could alleviate the growth inhibition of *Q. mongolica* seedlings under salt stress and relieve the symptoms of salt injury. The inoculation of the two fungi also promoted an increase in the cross-sectional area of the root system, Chl content, Pn, osmotic adjustment substances content, and antioxidant enzyme activity in seedlings. The effects of *G. viscidus* were stronger than those of *S. luteus* under salt stress. Generally, the results of our study underscore the need to screen for suitable ectomycorrhizal fungal species to inoculate *Q. mongolica* to enhance its salt resistance and, thus, ensure normal growth under salt stress.

## Figures and Tables

**Table 1:** The thickness, radius, and ratio of each part of the anatomical structure in the ECM formation period of *Q. mongolica*.

Treatment		Root Radius (μm)	Vascular Bundle Radius (μm)	Vascular Bundl Radius/Root Radius	Cortical Cells Thickness (μm)	Fungus Sheath Thickness (μm)
Control group	10 d	42.46 e	22.96 f	0.54 b	─	─
	20 d	44.74 e	24.08 e	0.54 b	─	─
	30 d	52.20 d	40.19 a	0.77 a	─	─
*G. viscidus* group	10 d	70.34 c	24.91 e	0.35 c	47.58 c	16.43 a
	20 d	75.98 b	26.56 d	0.35 c	50.52 b	16.98 a
	30 d	81.27 a	27.42 cd	0.34 d	54.48 a	17.17 a
*S. luteus* group	10 d	77.37 b	26.37 d	0.34 cd	50.94 b	14.31 b
	20 d	79.99 ab	28.41 c	0.36 c	51.62 b	17.81 a
	30 d	81.88 a	30.93 b	0.38 c	51.23 b	17.70 a

Note: Different lowercase letters within columns indicate significant differences at *p* < 0.05.
